# Paced segment characteristics predict clinical response to cardiac resynchronization therapy: results from the multimodality imaging assessment of pacing intervention in heart failure (MAPIT-HF) study

**DOI:** 10.1186/1532-429X-13-S1-O50

**Published:** 2011-02-02

**Authors:** Jorge A Wong, David Scholl, Raymond Yee, John Stirrat, Kris Carter, David McCarty, Nowell Fine, Andrew Krahn, Lorne Gula, Allan Skanes, Peter Leong-Sit, George Klein, Maria Drangova, James A White

**Affiliations:** 1 University of Western Ontario, London, ON, Canada

## Introduction

Cardiac Resynchronization Therapy (CRT) has been shown to improve quality of life and decrease mortality in heart failure patients. However, up to 40% of patients fail to respond to this therapy. Validation of a response prediction model that incorporates both myocardial scar and dyssynchrony of the paced myocardial segments would allow for a targeted approach to CRT lead delivery.

## Methods

Patients planned for CRT under standard indications were prospectively enrolled. Serial short-axis tagged cine and delayed enhancement MRI was performed using standard imaging protocols. Echocardiography and gated CT Angiography (CTA) were performed at baseline and 3 months post-implantation. The repeat CTA was performed for accurate lead tip localization to a 16-segment model. Dyssynchrony was measured for each segment (time to maximal radial strain, Trs) from serial short-axis tagged MRI and expressed in milliseconds from onset of pulse trigger (InTag, OsirX). A segmental scar score was then assigned using a blinded visual interpretation (score 0 to 4). The number of response prediction rules met was determined for each patient as follows: 1) LV lead tip placed on a dyssynchronous segment (Trs > 130msec), 2) LV lead tip placed on a viable segment (scar score <2), 3) RV lead tip placed on a viable segment (scar score <2). Clinical response to CRT, defined as a >=15% reduction in LVESV by echocardiography, was correlated to the number of prediction rules met.

## Results

Forty consecutive patients were enrolled with a mean age and ejection fraction of 67.0 ± 8.6 years and 25.6 ± 6.6%, respectively. Twenty four patients (60%) met clinical response criteria with a mean reduction in LVESV of 20.5 ± 16.5% compared to a rise of 1.4 ± 6.3% in non-responders (p<0.001). A strong correlation was seen between the number of prediction rules met and clinical response to CRT. In patients with 3, 2, 1 and 0 prediction rules met, the response rates were 100%, 92%, 58% and 40%, respectively (p<0.001). A strong correlation was also seen between the number of prediction rules and the mean reduction in LVESV [28%, 14% and 5% reduction in those with 3, 2 and 1 rules met, respectively (p=0.002)].

## Conclusions

Dyssynchrony and scar characteristics of the paced myocardial segments are strongly correlated with clinical response to CRT. A simple 3-point prediction model incorporating these variables appears to be highly predictive of response, and may be valuable for the selection of optimal pacing targets for CRT.

